# Using multiple data types and integrated population models to improve our knowledge of apex predator population dynamics

**DOI:** 10.1002/ece3.3469

**Published:** 2017-10-11

**Authors:** Florent Bled, Jerrold L. Belant, Lawrence J. Van Daele, Nathan Svoboda, David Gustine, Grant Hilderbrand, Victor G. Barnes

**Affiliations:** ^1^ Carnivore Ecology Laboratory Mississippi State University Mississippi State MS USA; ^2^ Kodiak Wildlife Services Kodiak AK USA; ^3^ Alaska Department of Fish and Game Kodiak AK USA; ^4^ United States Geological Survey Alaska Science Center Anchorage AK USA; ^5^ Grand Teton National Park National Park Service Moose WY USA; ^6^ National Park Service Alaska Regional Office Anchorage AK USA; ^7^ Kodiak Brown Bear Trust Westcliffe CO USA

**Keywords:** Bayesian, brown bear, hierarchical modeling, Integrated population model, Kodiak Island, *Ursus arctos*

## Abstract

Current management of large carnivores is informed using a variety of parameters, methods, and metrics; however, these data are typically considered independently. Sharing information among data types based on the underlying ecological, and recognizing observation biases, can improve estimation of individual and global parameters. We present a general integrated population model (IPM), specifically designed for brown bears (*Ursus arctos*), using three common data types for bear (*U*. spp.) populations: repeated counts, capture–mark–recapture, and litter size. We considered factors affecting ecological and observation processes for these data. We assessed the practicality of this approach on a simulated population and compared estimates from our model to values used for simulation and results from count data only. We then present a practical application of this general approach adapted to the constraints of a case study using historical data available for brown bears on Kodiak Island, Alaska, USA. The IPM provided more accurate and precise estimates than models accounting for repeated count data only, with credible intervals including the true population 94% and 5% of the time, respectively. For the Kodiak population, we estimated annual average litter size (within one year after birth) to vary between 0.45 [95% credible interval: 0.43; 0.55] and 1.59 [1.55; 1.82]. We detected a positive relationship between salmon availability and adult survival, with survival probabilities greater for females than males. Survival probabilities increased from cubs to yearlings to dependent young ≥2 years old and decreased with litter size. Linking multiple information sources based on ecological and observation mechanisms can provide more accurate and precise estimates, to better inform management. IPMs can also reduce data collection efforts by sharing information among agencies and management units. Our approach responds to an increasing need in bear populations’ management and can be readily adapted to other large carnivores.

## 1 INTRODUCTION

Large carnivores have great ecological, cultural, and economic value (Kellert, Black, Rush, & Bath, 1996; Ray, Redford, Steneck, & Berger, 2013). These species help maintain ecosystem function and stabilize interactions between species at lower trophic levels through predation and scavenging (Miller et al., 2001; Ripple et al., 2014). Large carnivores also represent a valuable resource for ecotourism and hunting (Sillero‐Zubiri & Laurenson, 2001). However, interactions between wild animals and the public can also result in negative outcomes for involved humans, animals, and financial institutions (Treves & Karanth, 2003). Maximizing positive aspects of human–large carnivore interactions, while minimizing human–wildlife conflicts, relies on effective management policies.

Population monitoring is an integral part of management, providing valuable information to assess management actions and devise new strategies. It relies on various metrics, sampling designs, and data types. Metrics commonly used for large carnivore management include density, abundance, and distribution (Taylor & Lee, 1995; Apps, McLellan, Woods, & Proctor, 2004; Gardner, Royle, Wegan, Rainbolt, & Curtis, 2010) with associated policies targeting the improvement or control of population parameters such as survival, persistence or colonization, reproductive rates, or connectivity (Noss, Quigley, Hornocker, Merrill, & Paquet, 1996; Ferreras, Gaona, Palomares, & Delibes, 2001). To assess these metrics, counts, detections, and reproductive success can be recorded using multiple sampling designs (e.g., repeated surveys, distance sampling, and capture–mark–recapture) (Gese, 2001; Hansen, Frair, Underwood, & Gibbs, 2015). As large carnivore populations often cross jurisdictional boundaries, populations of conservation interest are often studied simultaneously by different organizations using varying approaches. Count data (repeated or not) are common, logistically simple to collect, and are cost‐effective for investigating population statuses and trajectories. In contrast, mark‐recapture data are effective for providing information regarding demographic rates, but are less cost‐effective and resource‐intensive. Consequently, depending on the objectives of different studies and the resources available to different organizations or agencies, separate data sampling designs might be implemented on the same area or population. These different approaches and data sources are often considered independently and can result in underutilization of information and resources, with weaker estimates than if considered jointly.

Recently developed integrated population models (IPM) offer a framework to jointly analyze multiple demographic data and provide a unified approach incorporating population count data (i.e., data on population size) and demographic data (i.e., data on demographic rates) (Schaub & Abadi, 2011). In their most basic form, IPMs combine abundance analysis of count data (e.g., through state‐space models) with estimation of demographic parameters from capture–recapture models and marked individuals (Chandler & Clark, 2014). Advantages of these models include the estimation of more demographic parameters with greater precision and improved consideration of sources of uncertainty related to ecological and observation processes of each data type (Besbeas, Freeman, Morgan, & Catchpole, 2002; Gauthier, Besbeas, Lebreton, & Morgan, 2007; Abadi, Gimenez, Arlettaz, & Schaub, 2010). They also offer a framework to combine data from different surveys carried out on large areas or with temporally varying resources (conditions for which low‐intensity surveys are often more convenient). The integrated population modeling approach provides a meaningful description of ecological processes and a powerful tool to better understand demographic changes in large carnivore populations, while integrating multiple data sources.

Brown bears (*Ursus arctos*) present a management challenge as they are a long‐lived species with considerable individual variation (e.g., habitat, diet, and reproduction traits; Gillies et al., 2006; Edwards, Derocher, Hobson, Branigan, & Nagy, 2011; Lafferty, Belant, & Phillips, 2015). Brown bears also traverse large areas and commonly cross jurisdictional boundaries of agencies with different missions and mandates. Consequently, understanding the relationships between temporal, spatial, and environmental factors and brown bear demographics (abundance, distribution, and dynamics) is essential for effective management. Unfortunately, decisions can be constrained due to a lack of integrated data that would allow managers to more feasibly and robustly assess the status of brown bear populations. Integrated population models allow the estimation of parameters and identification of factors important for management that a single data source cannot estimate.

Factors affecting bear populations can broadly be categorized as biological, environmental, and anthropogenic, and to a lesser extent, genetic and random. These factors directly influence population dynamics by impacting different parameters such as reproductive success or survival of adults and young (Schwartz, Haroldson, & Cherry, 2006; Schwartz, Haroldson, & White, 2006b). For example, biological factors affecting survival probabilities of brown bears include age and sex (McLellan et al., 1999; Schwartz, Miller, & Haroldson, 2003; Schwartz et al., 2006b), as well as mother's age and litter size for young (McLaughlin, Matula, & O'Connor, 1994; Craighead, Sumner, & Mitchell, 1995; Mattson, 2000; Schwartz et al., 2006, 2006b). One of the most important ecological factors affecting brown bear populations is food quality and availability, which directly affects home range size, habitat use, and population density through survival and reproductive success (Hilderbrand et al.,^1999^). One of the most direct and visible ways humans impact bear populations is through harvest (Pease & Mattson, 1999; Boyce, Blanchard, Knight, & Servheen, 2001; Haroldson, Schwartz, & White, 2006). The impact of these factors (biological, environmental, anthropogenic, genetic, and random) can be detected in different, but complementary, data types. Because they impact reproductive success, effects of age or food availability can be estimated through repeated population counts (e.g., yearly counts with multiple replicates per year) and litter information. Similarly, as harvest and sex affect individual survival probabilities, these factors can be studied using repeated counts and capture–recapture data. Biological, ecological, and observational linkages among data types can then be exploited to better assess the importance of these factors.

**Figure 1:**
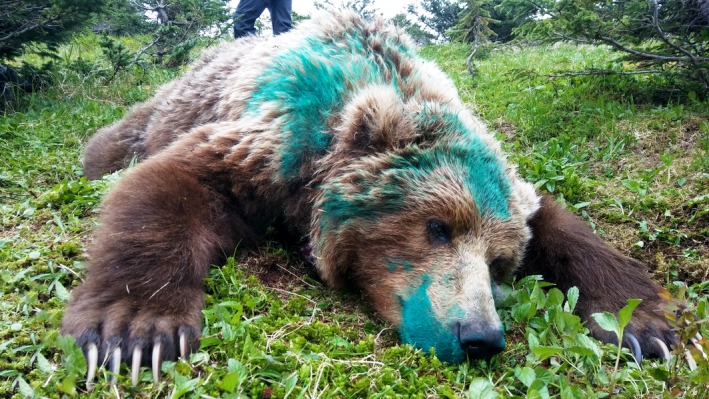
Kodiak brown bear (*Ursus arctos* middendorffi). This induced sow has been collared before release. Green ink was used to tattoo lips and spread on the head and shoulders to allow for quick identification of recently captured animals and prevent unnecessary early recapture

We present a general Integrated Population Model, specifically targeted for management of brown bears, explicitly connecting repeated counts, capture–mark–recapture, and litter information using biological, ecological, and observational relationships. We expected this approach to improve precision and accuracy of population estimates. We tested these assumptions using a simulated population. We then present an application of this general approach adapted to the constraints of a case study using historical long‐term demographic data for Kodiak brown bears (*U. a. middendorffi*, Figure 1) on Kodiak Island, Alaska, USA. We compared results from our model to the extensive literature on this population to evaluate the practical application of our model.

## 2 MATERIALS AND METHODS

The hierarchical Bayesian framework allows for a joint modeling of different data types suitable for integrated population models, using an explicit description of the mechanisms responsible for these data. It also allows integration of information from literature or expert opinion with the inclusion of informative priors for relevant parameters.

**Figure 2:**
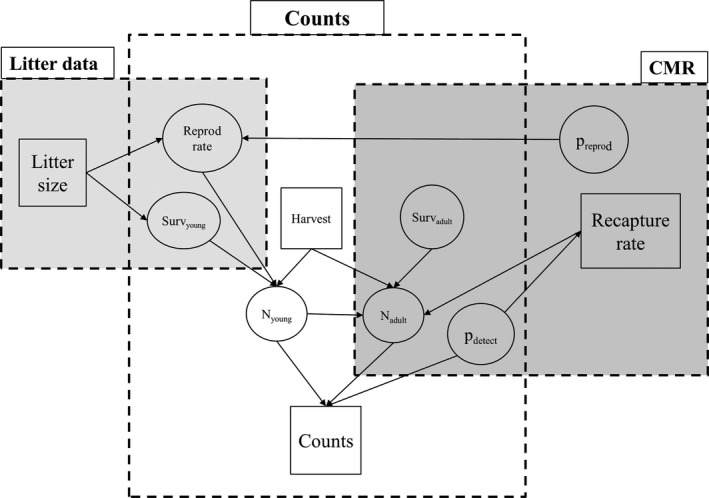
Links among three data types (dashed boxes) used for the integrated population model for brown bears, based on available data (boxes), and derived parameters (circles). With Surv: survival probabilities, *p*_reprod_: reproduction probability, *N*: abundance, and *p*_detect_: detection probability

In the general modeling approach for large carnivores populations, and brown bears in particular, the most commonly available data are repeated counts (including harvest data, defined as anthropogenic mortality), capture–mark–recapture (CMR) data, litter size, and radio telemetry data (e.g., Miller et al., 1997; Barnes & Smith, 1998; Solberg, Bellemain, Drageset, Taberlet, & Swenson, 2006). Counts can be used to assess the size of a target population, potentially differentiating between different age and sex classes, to better understand the composition and dynamics of said population. CMR data can provide more detailed information about individual survival and reproductive success. Information on litters can often be collected at the same time as capture–recapture data to estimate survival of young and adult reproductive success. In this context, these distinct data types can be linked through ecological and observation components. Ecological process refers to the underlying true state of the population, and the factors and mechanisms affecting this state; the observation processes will reflect the partial access we have to the underlying ecological process due to limitations emerging from “imperfect” observation methods. We developed a conceptual model to explicitly link these three common data types, specifically targeted for brown bears (Figure 2). This model offers a general framework for managers and can be used with modifications to fit their specific needs and available data. As an illustration of how this could be practically implemented, we also present a subset model for a case study where not all data are available.

### 2.1 General model description: Counts

N‐mixture models provide a convenient framework to analyze repeated counts while accounting for imperfect detection (Kery, Royle, & Schmid, 2005; Royle, 2004). Following this approach, we assumed repeated count data collected across years (i.e., yearly counts with within‐year replicates). For any given sex–age class *X* during replicate *k* in year *t*, we can define the set of repeated counts $C_{X_{t},k}$ following a binomial distribution:

$$C_{X_{t},k}∼\text{Binomial}\left(N_{X_{t}},p\right)$$

where $N_{X_{t}}$ corresponds to the actual population size for the sex–age class *X* during year *t*, and *p* represents the individual detection probability (i.e., probability that an individual available for detection is actually detected). For simplicity, we set *p* as constant over time and classes, but this assumption can trivially be removed.

Subsequent definition of the corresponding sex–age class populations is done using a simple population model where each class size is defined based on the structure of the general population. We used five age classes (cub [<1 year old], yearling [1 year old], dependent young [>1 year old but with adult female], subadult [3‐4 years old], and adult [>4 years old]). Because we know that survival probabilities differ between sexes (McLellan et al., 1999; Schwartz et al., 2003, 2006b), we further divided each age class into male and female classes. In the following equations, these age classes are referred to as subscripts C, Y, D, S and A, respectively. For brevity, only equations for females are presented, and male age class population size is defined similarly. Global cub population size $N_{C_{t_{\mathrm{BH}}}}$ in year *t*, before harvest (BH), can be defined as following a Poisson distribution such as:

$$N_{C_{t_{\mathrm{BH}}}}∼\text{Poisson}\left(N_{A{♀}_{t-1}}\Phi_{A{♀}_{t-1}}P_{t-1}L_{t-1}\right)$$

where the Poisson mean is the product of total adult female abundance in year $t-1\;N_{A{♀}_{t-1}},$ that survived and reproduced from year *t‐1* to year *t* with probabilities $\Phi_{A{♀}_{t-1}}$ and $P_{t-1}$, respectively, and the average litter size *L*_*t*−1_ at den exit between years *t*−1 and *t*. This represents cubs born between years *t*−1 and *t* surviving until den exit. Consequently, male and female cub abundance during year *t* before harvest ($N_{C{♂}_{t_{\mathrm{BH}}}}$ and $N_{C{♀}_{t_{\mathrm{BH}}}}$, respectively) is defined based on the global cub population size, and the corresponding litter sex ratio following

$$\begin{array}{cc} & N_{C{♂}_{t_{\mathrm{BH}}}}∼\text{Binomial}\left(N_{C_{t_{\mathrm{BH}}}},♂:{♀}_{t-1}\right)\\ & N_{C{♀}_{t_{\mathrm{BH}}}}=N_{C_{t_{\mathrm{BH}}}}-N_{C{♂}_{t_{\mathrm{BH}}}} \end{array}$$

from which, we can define the total abundances for male and female cubs $N_{C{♂}_{t}}$ and $N_{C{♀}_{t}}$ in year *t*, after harvest. For example, for females:

$$N_{C{♀}_{t}}=N_{C{♀}_{t_{\mathrm{BH}}}}-H_{C{♀}_{t}}$$

(1)with $H_{C{♀}_{t}}$ the number of female cubs harvested in year *t* (typically equal to 0). We assume here that information about harvest is available and observed without error. If not, a separate modeling effort should be carried out to account for incomplete harvest reporting, or interpretation about survival probabilities should acknowledge that they include in part anthropic mortality.

Male and female yearling populations in year *t*, before harvest (BH) ($N_{Y{♂}_{t_{\mathrm{BH}}}}$ and $N_{Y{♀}_{t_{\mathrm{BH}}}}$, respectively), are defined based on the corresponding cubs population and sex‐specific survival probabilities in year *t‐1* ($\Phi_{C{♂}_{t-1}}$ and $\Phi_{C{♀}_{t-1}}$). Actual male and female yearling populations in year *t*, after harvest ($N_{Y{♂}_{t}}$ and $N_{Y{♀}_{t}}$), are then computed by accounting for their respective harvest results ($H_{Y{♂}_{t}}$ and $H_{Y{♀}_{t}}$) following equation 1. For example, for females

$$N_{Y{♀}_{t_{\mathrm{BH}}}}∼\text{Binomial}\left(N_{C{♀}_{t-1}},\Phi_{C{♀}_{t-1}}\right)$$

Dependent young abundances $N_{D{♂}_{t_{\mathrm{BH}}}}$ and $N_{D{♀}_{t_{\mathrm{BH}}}}$ at year *t*, before harvest, can be defined in terms of yearlings that survived in year *t*−1 to become dependent young and dependent young that survived in year *t‐1* but did not wean. For example, for females:

$$N_{D{♀}_{t_{\mathrm{BH}}}}∼\text{Binomial}\left(N_{D{♀}_{t-1}},\Phi_{D{♀}_{t-1}}\left(1-\gamma_{D{♀}_{t-1}}\right)\right)+\text{Binomial}\left(N_{Y{♀}_{t-1}},\Phi_{Y{♀}_{t-1}}\right)$$

with $\Phi_{Y{♀}_{t-1}}$ yearling survival probabilities, and $\Phi_{D{♀}_{t-1}}$ dependent young survival probabilities for females. Transition rates from dependent young to subadults, indicating successful weaning, correspond to $\gamma_{D{♀}_{t-1}}$. It should be noted that the above notation does not refer to a mixture distribution, but rather indicates that dependent abundance before harvest is the sum of two independent binomial random variables: abundance of yearlings that survived and became dependent young and abundance of dependent young that survived but did not yet reach independence. This notation has been adopted in the rest of the article for brevity. The actual abundances of dependent young ($N_{D{♂}_{t}}$ and $N_{D{♀}_{t-1}}$) at year *t*, once harvest has been taken into account (reported as $H_{D{♂}_{t}}$ for males, and $H_{D{♀}_{t}}$ for females), are then derived following equation 1.

Similarly, the subadult population is the result of subadults surviving between year *t‐1* and year *t* not transitioning to adult, of the surviving dependent young that actually weaned to become subadults, and of yearly harvest $\left(H_{S{♂}_{t}}\;\mathrm{and}\;H_{S{♀}_{t}}\right)$ (following Equation 1). For example, for females, this can be described as:

$$N_{S{♀}_{t_{\mathrm{BH}}}}∼\text{Binomial}\left(N_{S{♀}_{t-1}},\Phi_{S{♀}_{t-1}}\left(1-\gamma_{S{♀}_{t-1}}\right)\right)+\text{Binomial}\left(N_{D{♀}_{t-1}},\Phi_{D{♀}_{t-1}}\gamma_{D{♀}_{t-1}}\right)$$

where $N_{S{♀}_{t_{\mathrm{BH}}}}$ and $N_{S{♀}_{t}}$ are female subadult abundance before and after harvest during year *t*, $\Phi_{S{♀}_{t-1}}$ is the subadult survival probability between year *t‐1* and year *t*, and $\gamma_{S{♀}_{t-1}}$ the probability that surviving subadults will transition to adult. The corresponding counterparts for male subadults are $N_{S{♂}_{t_{\mathrm{BH}}}}$, $N_{S{♂}_{t}}$, $\Phi_{S{♂}_{t-1}}$, and $\gamma_{S{♂}_{t-1}}$, respectively.

Adult populations before harvest ($N_{A{♂}_{t_{\mathrm{BH}}}}$ and $N_{A{♀}_{t_{\mathrm{BH}}}}$) on year *t* are based on adult and subadult populations the previous year (after harvest), taking into account adult survival probabilities ($\Phi_{A{♂}_{t-1}}$ and $\Phi_{A{♀}_{t-1}}$) and subadult survival and transition probabilities. For example, for females

$$N_{A{♀}_{t_{\mathrm{BH}}}}∼\text{Binomial}\left(N_{S{♀}_{t-1}},\Phi_{S{♀}_{t-1}}\gamma_{S{♀}_{t-1}}\right)$$

Final adult populations at year *t* ($N_{A{♂}_{t}}$ and $N_{A{♀}_{t}}$) are then obtained after taking into account harvest number for both males and females ($H_{A{♂}_{t}}$ and $H_{A{♀}_{t}}$) following equation 1.

A detailed diagram of the general population model is presented in Appendix S1.

### 2.2 General model description: CMR

Capture–mark–recapture data (e.g., Jolly‐Seber, Cormack‐Jolly‐Seber, robust designs, Williams, Nichols, & Conroy, 2002) are often collected for large carnivores, including brown bears, to monitor population size, growth rate, and/or vital rates responsible for changes in this state variable. We present here a modeling approach similar to a CJS model including dead recovery, but this could easily be modified to accommodate different designs. For a general IPM where CMR data for brown bears are available over several years, we define detection *y*_*i,t*_ for bear *i*, during year *t* conditionally on the individual actual alive status *z*_*i,t*_. We can detect an individual as recovered/seen alive (*z*_*i,t*_ = 1) or dead (*z*_*i,t*_ = 0). If the individual is detected alive, *y*_*i,t*_ is equal to 1, and to 0 otherwise. Because of how records are typically kept, we included a third variable *r*_*i,t*_ where *r*_*i,t*_ = 1 indicates that the individual has been recovered dead (while *r*_*i,t*_ = 0 corresponds to an individual that is still alive, or dead but not recovered). Once an individual has been recovered dead, its recapture history ends, effectively restricting the recapture vectors *y*_*i,t*_ and *r*_*i,t*_. up to the year of detected death. In practice, this allows us to consider two separate detection probabilities *p* and *p′*, for individuals alive or dead, respectively. We note that if CMR and count data are collected using the same general protocol, it would be possible to assume that their detection probability is the same. We then formally defined survival status, detection, and dead recovery, using Bernoulli distribution such as:

$$\left\{\begin{array}{c} z_{i,t}∼\text{Bernoulli}\left(z_{i,t-1}\varphi_{i,t-1}\right)\\ y_{i,t}∼\text{Bernoulli}\left(pz_{i,t}+p^{\prime }\left(1-z_{i,t}\right)\right)\\ r_{i,t}∼\text{Bernoulli}\left(p^{\prime }\left(1-z_{i,t}\right)\right) \end{array}\right.$$

where detectability and dead recovery are defined conditionally on the survival status in year *t*. Survival of individual *i* in year *t* is conditional on its status during the previous year, and its specific survival probability *ϕ*_*i*,*t*−1_ between year *t*−*1* and *t*. Subsequently, the survival probability can be expressed as a linear combination on the logit scale of an intercept β_*z*_, some relevant covariates *x*_*z*_ (time‐individual dependent or not) and their associated slopes α_*z*_, and optionally a random effect $\varepsilon_{z_{i,t}}$.

$$\text{logit}\left(\varphi_{i,t}\right)=\beta_{z}+\sum \alpha_{z}x_{z}+\varepsilon_{z_{i,t}}$$

Supplementary information about the detected reproductive status *R*_*i,t*_ of female individual *i* during year *t* can be recorded (with *R*_*i,t*_ = 1 if individual *i* successfully reproduced in year *t*, 0 otherwise) and modeled following a Bernoulli distribution:

$$R_{i,t}=\text{Bernoulli}\left(\rho_{i,t}p_{r}z_{i,t}\right)$$

with ρ_*i,t*_ being the probability that individual *i* successfully reproduced in year *t* (i.e., is accompanied by cubs), and *p*_*r*_ the specific detectability of the reproductive status. This effectively conditions the detection of the reproductive status on reproductive rates, detectability, and survival of the individual. As for the individual survival probability, we can express the successful reproduction probability as a linear combination on the logit scale of an intercept β_*r*_, any relevant covariates *x*_*r*_ (time‐individual dependent or not) and their associated slopes α_*r*_, and optionally a random effect $\varepsilon_{r_{i,t}}$:

$$\text{logit}\left(\rho_{i,t}\right)=\beta_{r}+\sum \alpha_{r}x_{r}+\varepsilon_{r_{i,t}}$$

### 2.3 General model description: Litter

Information about the reproductive success of individuals is often obtained simultaneously to population counts or CMR data. We can distinguish two essential elements of information: initial litter size ℓ_*k*,1_ for a litter *k*, and changes in litter size over time (due to mortality). Females will often care for their young for periods of 1.4 to 2.5 years before reproducing again (Hensel, Troyer, & Erickson, 1969; Dahle & Swenson, 2003). Weaning age can be recorded and provide useful information about the structure of the population (e.g., ratio of dependent young to subadults, parental investment). Initial litter size at den exit can be modeled following a Poisson distribution with mean λ_*k*_; the Poisson mean can then be defined on the log scale as a linear combination of an intercept β_*L*_, any relevant factor *x*_*L,*_ and their corresponding slopes α_*L*_, and if necessary a random effect $\varepsilon_{L_{k}}$.

$$\ell_{k,1}∼\text{Poisson}\left(\lambda_{k}\right)$$

$$\log\left(\lambda_{k}\right)=\beta_{L}+\sum \alpha_{L}x_{L}+\varepsilon_{L_{k}}$$

The litter size in subsequent years can be described dynamically using a binomial distribution with litter size the previous year as sample size, and young survival probabilities between year *t* *− 1* and *t* ($\varphi_{{\mathrm{young}}_{k,t-1}}$) as success probability, such as:

$$\ell_{k,t}∼\text{Binomial}\left(\ell_{k,t-1},\varphi_{{\mathrm{young}}_{k,t-1}}\right),$$

where the survival probability is defined on the logit scale as a linear combination of an intercept β_young_, any relevant factor *x*_young*,*_ and their corresponding slopes α_young_, and if necessary a random effect $\varepsilon_{{\mathrm{young}}_{k}}$:

$$\text{logit}\left(\varphi_{{\mathrm{young}}_{k,t-1}}\right)=\beta_{\mathrm{young}}+\sum \alpha_{\mathrm{young}}x_{\mathrm{young}}+\varepsilon_{{\mathrm{young}}_{k}}$$

The probability that a litter weans (i.e., *W*_*k*_ = 1, 0 otherwise) is defined conditionally on the age of the litter. Following what is available in literature (e.g., Hensel et al., 1969; Dahle & Swenson, 2003), we assumed that weaning does not occur if young are less than 2 years old, and that litters 4 years old or greater have weaned. Litters 2 and 3 years old will have a probability of weaning $p_{w_{k,t}}$:

$$W_{k}=\left\{\begin{array}{cc} 0 & \text{if age}\;<2\\ \text{Bernoulli}\left(p_{w_{k,t}}\right) & \text{if}\;2⩽\text{age}⩽3\\ 1 & \text{if age}⩾4 \end{array}\right.$$

As described previously, we can further our description of the weaning probability using a linear combination on the logit scale of an intercept (β_*w*_), covariates (*x*_*w*_), and corresponding slopes (α_*w*_) if relevant and sufficient data are available, and if useful a random effect ($\varepsilon_{w_{k}}$).

$$\text{logit}\left(p_{w_{k,t}}\right)=\beta_{w}+\sum \alpha_{w}x_{w}+\varepsilon_{w_{k}}$$

### 2.4 General model description: Links

Once the model used to describe each individual data type has been set, we can establish links allowing for information sharing between datasets (Figure 2; Schaub & Abadi, 2011). While data collected at the individual level through CMR reflect individual histories and account for individual variation (e.g., age or location), this information needs to be summarized among multiple individuals to reflect global processes at the population scale. This approach assumes that the CMR data represent an accurate sample of the entire population and the general range of factors that affect it. This assumption is necessary to consider that the average of the demographic parameters over the individual scale (individual survival and reproduction probabilities) provides an accurate estimate of the survival and reproduction probabilities at the scale of the whole population. The global female reproduction probability *P*_*t*_ for counts can be informed by the average individual reproduction probability of females during year *t* ($n_{\rho_{t}\;\text{: total number of adult female alive during year}\;t})$, using CMR data, such as:

$$P_{t}=\frac{1}{n_{\rho_{t}}}\sum \rho_{i,t}z_{i,t}$$

Following the same approach, CMR data can also be used to inform count modeling through refining the global yearly survival probabilities of adults and subadults by averaging the survival probability of individuals alive the previous year in function of age‐sex classes:

$$\begin{array}{cc} & \left\{\begin{array}{c} \Phi_{S{♂}_{t}}=\frac{1}{n_{S{♂}_{t}}}\sum_{S♂}\varphi_{i,t}z_{i,t}\\ \Phi_{A{♂}_{t}}=\frac{1}{n_{A{♂}_{t}}}\sum_{A♂}\varphi_{i,t}z_{i,t} \end{array}\right.\\ & \left\{\begin{array}{c} \Phi_{S{♀}_{t}}=\frac{1}{n_{S{♀}_{t}}}\sum_{S♀}\varphi_{i,t}z_{i,t}\\ \Phi_{A{♀}_{t}}=\frac{1}{n_{A{♀}_{t}}}\sum_{A♀}\varphi_{i,t}z_{i,t} \end{array}\right. \end{array}$$

Litter information can be used similarly to provide more detailed information about young survival. As with the above formulation, we can average the individual survival probabilities in litters as a function of their age and sex to model the global yearly survival probability of cubs, yearlings, and dependent young ($\Phi_{C{♂}_{t}}$ and $\Phi_{C{♀}_{t}}$, $\Phi_{Y{♂}_{t}}$ and $\Phi_{Y{♀}_{t}}$, $\Phi_{D{♂}_{t}}$ and $\Phi_{D{♀}_{t}}$, respectively, for males and females). We assume that information on sex of young is not available during dependence; this assumption could easily be removed if this information is available.

Similarly, the global yearly litter size *L*_*t*_ for counts can be expressed as the average size of all the individual litters born in year *t*.

### 2.5 Priors

When additional information from which parameters can be derived is unavailable, as shown above, we can use informative or noninformative priors. For the general count model, we included transition rates (from dependent young to subadults $\gamma_{S{♂}_{t}}$ and $\gamma_{S{♀}_{t}}$, and from subadults to adults $\gamma_{A{♂}_{t}}$ and $\gamma_{A{♀}_{t}}$) with noninformative priors, such as for example:

$$\gamma_{S{♂}_{t}}∼\text{Uniform}\left(0,1\right)$$

It can be beneficial to use information from the literature or expert opinion to develop informative priors. For example, the sex ratio could be provided with a noninformative prior and be estimated from count and litter information. However, as abundant literature is available for brown bears, we used an informative Beta prior, such as:

$$♂:{♀}_{t}∼Beta\left(a,b\right)$$

with

$$a=\frac{\mu_{♂:♀}^{2}\left(1-\mu_{♂:♀}\right)-\mu_{♂:♀}\sigma_{♂:♀}^{2}}{\sigma_{♂:♀}^{2}}\;\text{and}\;b=\frac{a(1-\mu_{♂:♀})}{\mu_{♂:♀}},$$

where $\mu_{♂:♀}$ and $\sigma_{♂:♀}^{2}$ correspond to estimated mean and variance for the sex ratio, from the literature. This is derived from the relationship between the beta distribution's mean and variance with its shape parameters:

$$\begin{array}{cc} \mu_{♂:♀} & =\frac{a}{a+b}\\ \sigma_{♂:♀}^{2} & =\frac{ab}{{\left(a+b\right)}^{2}\left(a+b+1\right)} \end{array}$$

For Kodiak island, we set $\mu_{♂:♀}=0.5238$ and $\mu_{♂:♀}=0.0771,$ using information from Troyer and Hensel (1964).

Finally, if we assume the individual detection probability is the same between data collected during the count survey and the CMR data, we can set a noninformation prior and use information from count and CMR data to determine the parameter's posterior distribution.

$$p∼\text{Uniform}\left(0,1\right)$$

If we assume that detectabilities for CMR and count data differ, separate priors for each detection probability should be used.

We also set a noninformative prior for the recovery probability (p’) and reproduction detectability (p_r_).

### 2.6 Simulations

To demonstrate the advantages of jointly using multiple data sources, we compared population estimates from the general integrated population model described above with population estimates from the counts section only of this model for increasing, decreasing, and stable populations. Randomly drawing sets of values for demographic parameters using intervals commonly estimated in the literature specific to coastal brown bears (e.g., Schwartz et al., 2006; Schwartz, Haroldson, & White, 2006a,b), we simulated populations following these different dynamics and created several datasets (counts, CMR, and litter information) reflecting ecologically realistic conditions allowing us to assess the accuracy of our model.

We simulated counts over 20 time units (years), with four replicates per year. Over the same time period, we simulated CMR data for 1,000 individuals, and data for 200 litters corresponding to a total of 30 reproducing females. Reproduction parameters were set to allow the average litter size to vary in an interval that matched what is available in the literature for brown bears, between one and four cubs per litter (McLellan, 1994). We used the same approach for survival parameters. We set the detection probability to 0.7, the reproduction detectability to 0.8, and the recovery probability to 0.9.

## 3 CASE STUDY: KODIAK BROWN BEAR POPULATION

Although theoretical datasets provide a good basis to assess the efficiency of the integrated population model approach, it is essential to also consider its practicality by applying this method to a less optimal situation such as what can be encountered in real‐life surveys where not all data are available.

We used data from 1983 to 1998 provided by the US Fish and Wildlife Service and Alaska Department of Fish and Game to model the dynamics of the Kodiak brown bear population. Kodiak island (9293 km^2,^ Meiri, Simberloff, & Dayan, 2005) is located in the western Gulf of Alaska (56°45′–58°00′N, 152°09′–154°47′W) and supports approximately 3,500 brown bears (Van Daele 2007). Kodiak Island has a subarctic maritime climate, with variable weather due to topographic relief (Van Daele, Barnes, & Smith, 1990). A detailed description of Kodiak Island vegetation can be found in Fleming and Spencer (2004). Despite this population being well‐studied, only partial data were available. CMR surveys and concomitant collection of litter information were regularly collected (Van Daele & Barnes, 2010), but not enough yearly count data were available for proper modeling. To demonstrate the versatility and adaptability of our IPM approach, we fitted a reduced version of our general model to these data, taking advantage of the connection between CMR reproduction data and litter information.

There were bear research projects on four primary study areas across Kodiak Island from 1982 to 1997, all of which included radio telemetry (Van Daele & Barnes, 2010). We used comparable capture, handling, and processing techniques in all investigations. For each captured bear, we noted gender, reproductive status, and extracted a first premolar for age determination (Matson et al., 1993). We deployed conventional VHF radio collar transmitters (Telonics Inc., Mesa, AZ, USA) on a sample of subadult and adult bears in each study area. The sample was purposefully biased toward adult females because they would provide the most information on productivity and cub survival, and because of concerns about neck injuries the collars could cause to subadults and males. Collared bears were typically radiotracked from a fixed‐winged aircraft (Piper PA‐18 or equivalent) weekly by experienced pilot/observer teams. We reduced the flight schedule to twice monthly during the winter months. Tracking flight frequency was increased during spring emergence to ascertain cub production and survival. CMR data consisted of yearly detections (i.e., whether or not a bear was successfully detected at least once during radiotracking in a given year) of a total of 241 marked bears (55 males and 186 females) during 1983–1998, representing 977 total detections. We recorded age, sex, and reproductive status (presence and age of dependent young) for each individual. Litter data were composed of an initial assessment of the litter size of the reproductive females resighted every year before den entrance. We also recorded a yearly follow‐up to determine if any young died and when weaning occurred. We were unable to collect information on sex ratios of litters. We collected data on 519 litters, representing 910 litter‐years (mean ± *SD* = 1.75 year per litter ±1.11).

We conducted a literature review of factors potentially impacting brown bear populations (Table 1) and selected a subset of parameters for which we had data. We retained eight covariates: age, sex, salmon availability (annual estimates of salmon biomass, Alaska Department for Fish and Game data, Van Daele, Barnes, & Belant, 2012), age of first reproduction, litter size, mother's age, presence of dependent young, and subadult status. Specifically, we modeled adult survival as a function of age (including a quadratic term), sex, and food availability (more specifically salmon availability). We also included a separate effect for subadults and a random effect. We expressed individual reproduction probability as a linear function on the logit scale of age, food availability, age of first reproduction, presence of dependent young, and included a random effect. Initial litter size was modeled as a function of the mother's age, its age of first reproduction, food availability (through salmon production), and a random effect. Covariates considered to potentially impact young survival probability were the young's age (including a quadratic effect), litter size, mother's age, food availability, and a random effect. Finally, we considered weaning probability to be solely dependent on the young's age and a random effect.

**Table 1:** Potential factors influencing brown bear population dynamics

Category	Factors	Survival			Reproductive success
		Cubs/yearlings/subadults	Adult males	Adult females	
Biological	Age	**+**	**+/‐**	**+/‐**	**+/‐**
	Sex	**♀ > ♂**	**−**	**+**	na
	Litter size	**+**	na	na	**+**
	Mother's age	**+**	na	na	na
	Presence of dependent young	na	na	na	**−**
	Age of first reproduction	na	na	na	**−**
	Perinatal mortality	**−**	na	na	na
	Interbirth interval	na	na	na	+
	Disease	**−**	**−**	**−**	na
	Intraspecific predation	**−**	na	na	**−**
Ecological	Food availability/Salmon stream density	**+**	**+**	**+**	**+**
	Habitat type/Forest cover	**+**	**+**	**+**	**+**
	Density	**−**	**−**	**−**	**−**
	Climate change	**−**	**−**	**−**	**−**
	Extreme weather	**+/−**	**−**	**−**	na
Anthropogenic	Harvest/Hunting	+/**−**	**− −**	**−**	na
	Management policies	**+/−**	**+/−**	**+/−**	**+/−**
	Human presence	**−**	**−**	**−**	**−**

Cells in dark gray with bold text correspond to essential interactions that should be considered. Cells with bold text only correspond to factors that should secondarily be explored. A “+”sign indicates an expected positive correlation between the population parameter and the factor; a “**−**”indicates an expected negative correlation; and “na” indicates there is either no relationship or that it is not relevant to our study.

## 4 IMPLEMENTATION

We implemented our models for both simulated and Kodiak Island datasets using program JAGS (Plummer, 2003), called from R (v.3.0.1, R Core Team, 2014) with the package *rjags* (Plummer, 2014; Appendix S2). We ran three chains using noninformative priors, for 50,000 iterations after a 50,000 iteration burn‐in (to insure convergence) with a thinning of 4. We monitored convergence by visual inspection of the MCMC chains and using the Gelman–Rubin convergence statistic (Gelman, Carlin, Stern, & Rubin, 2014). All results are presented with mean and 95% credible intervals.

## 5 RESULTS

### 5.1 Simulated datasets

**Figure 3:**
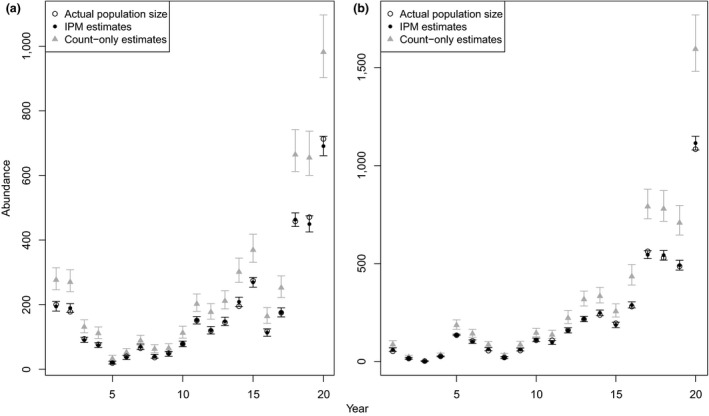
Comparison of estimates of a simulated population size over time using integrated population model (IPM) and replicated counts only. The left and right panels correspond to results for simulated adult male and subadult female subpopulations, respectively. Simulated abundances are indicated by a black circle, estimated abundances with corresponding 95% credible intervals are represented as black points for the IPM and gray triangles for the replicated counts only

Using simulated datasets, our integrated population model produced estimates extremely close to the actual simulated abundance of our theoretical populations, with estimates more accurate and precise than obtained using the count‐only component of the model (Figure 3). Credible intervals from the IPM included the true simulated population abundance 94% of the time whereas the count‐only model included the true population only 5% of the time. Considering that the underlying structure of the simulated dataset was similar to the one used by both models, the simple count model performance was less than expected. Population estimates from the count model were biased and imprecise (Figure 3).

### 5.2 Kodiak brown bear case study

**Figure 4:**
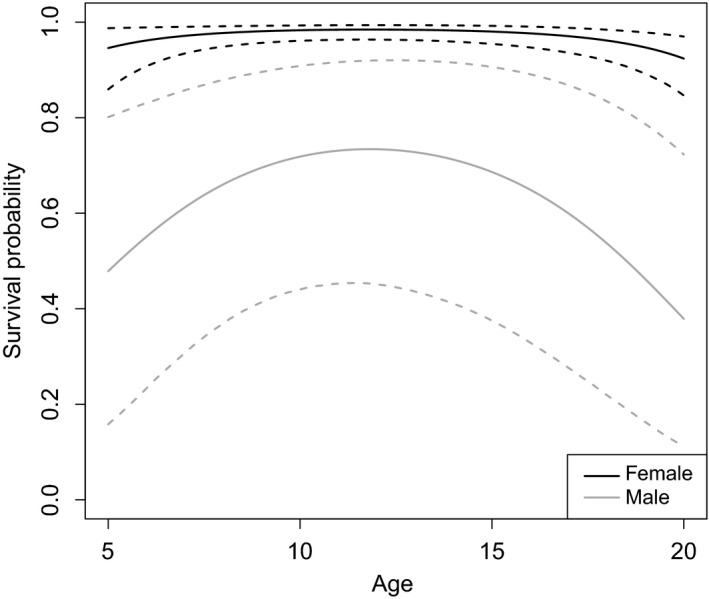
Survival probability as a function of age and sex for brown bears on Kodiak Island, Alaska, USA, 1983–1998. Mean (solid line) and 95% credible interval (dashed lines) are presented for adult males (gray) and females (black)

Adult survival probability differed between sexes (female having a higher survival probability, coefficient = 3.11 [1.72; 4.53]) and was correlated with food availability (slope = 0.68 [0.19; 1.21]). There was also a quadratic relationship between adult survival probability and age (quadratic slope = −0.03 [−0.04; −0.01], linear slope = 0.61 [0.18; 1.05]; Figure 4). We did not detect a subadult effect (coefficient = 0.25 [−2.18; 2.73]). Young survival probability increased with age: survival probability for cubs, yearlings, and dependent young were estimated as 0.89 [0.85; 0.92], 0.96 [0.94; 0.98], and 0.98 [0.96; 0.99], respectively. Young survival was negatively correlated with litter size (slope = −0.89 [−1.65; −0.24]). While we detected no correlation between reproduction probability and food availability (as expressed through salmon biomass, slope = −0.24 [−0.76; 0.21]), this probability was negatively correlated with age (slope = −0.43 [−0.69; −0.21]). The presence of dependent young ensured that a female had an effective reproduction probability of 0, with a coefficient on the logit scale of −89.01 [−230.32; −15.92]. This result suggests to fix the reproduction probability to 0 when dependent young are present for Kodiak brown bears. Estimated annual average litter size (within 1 year of birth) ranged from 0.45 [0.43; 0.55] to 1.59 [1.55; 1.82].

## 6 DISCUSSION

Our integrated population model provided estimates close to the simulated datasets, providing greatly improved accuracy and precision over population count model estimates. When applied to the Kodiak Island brown bear population dataset, the subset model provided results that were overall consistent with what is reported in the literature. The Integrated Population Model we present is not a Rube‐Goldberg machine or a black box, but an ecologically based approach to populations and communities that can be used to bridge the gap between ecology and management. Improving estimation with data that are often collected simultaneously, and therefore with minimal increase in cost, responds to a need for efficient and economical methods in wildlife management (Field, Tyre, & Possingham, 2005). The mechanistic description of ecological processes allows for a conceptual approach that can be adapted to different species in diverse ecosystems, and with different datasets, depending on objectives. The lack of some data can be compensated for using external available information (e.g., related scientific publications, external datasets). The Bayesian framework allows for easy integration of this information (Clark, 2005; Dupuis & Joachim, 2006; Morris, Vesk, & McCarthy, 2013).

Developing an IPM for brown bears required specific ecological model structures. Brown bears are solitary carnivores, with an omnivorous diet (Mowat & Heard, 2006; Bojarska & Selva, 2012). Reproducing females will often care for their young for several years before reproducing again (Dahle & Swenson, 2003). Males and females have different survival probabilities, and males are usually subject to more intense harvest pressure (McLellan et al., 1999; Bischof, Swenson, Yoccoz, Mysterud, & Gimenez, 2009; Van Daele & Barnes, 2010). Moreover, because bears are harvested in some jurisdictions, we need to explicitly incorporate harvest rates. Bear data, with populations being monitored using a large variety of sampling designs, methods, and metrics, will provide different information of varying quality. Information sharing among data types was largely responsible for the observed improvements in accuracy and precision of this IPM. We can improve the development of mechanistic models by considering how population trajectories are connected to individual histories.

Due to the particular history and geography of the region, the relationship between bears and humans in Kodiak is an especially interesting and challenging system to model. Interactions between Kodiak brown bears and the local communities have been complex and changing under the different pressures of human–bear conflicts, trophy hunting, and conservation efforts (Van Daele, 2003). We emphasized three of five broad categories of factors known to affect bear populations (biological, environmental, anthropogenic, genetic, and random). Our results regarding biological factors were overall consistent with previous studies, such as female survival being greater than that of males in North America (McLellan et al., 1999; Schwartz et al., 2003, 2006b) and survival of dependent young increasing with mother's age (McLaughlin et al., 1994; Mattson, 2000; Schwartz et al., 2006, 2006b). We also confirmed the importance of accounting for age to understand the drivers of population dynamics. We note that survival probability was overall high, especially for dependent young and adult females. Abundant high‐quality food, such as salmon in the Kodiak system, reduces competition for resources and facilitates improved condition and survival of these cohorts. The lower adult male survival could also be related to the fact that large males are more valuable hunting trophies (McLellan et al., 1999; Bischof et al., 2009). Reproduction probability on the other hand was negatively correlated with age, and a female would not reproduce if she were caring for dependent young. Interestingly, opposite to what we observed, cub and yearling survival have been shown to increase with litter size (Craighead et al., 1995; Schwartz et al., 2006b). Our litter size estimates and cub survival were respectively lower and higher than typically reported (Hensel et al., 1969; McLellan, 1994; Frković, Huber, & Kusak, 2001; Schwartz et al., 2003; Van Daele et al., 2012), possibly a consequence of cub mortality before we estimated litter size just before den entrance (about 4–6 months post den emergence), instead of at den emergence at the beginning of the season which is more typical.

Food availability can strongly influence aspects of brown bear ecology. Observed variation in home range and habitat use of brown bears on Kodiak Island largely reflected quality and availability of food, and bears had a demonstrated ability to successfully use a variety of food sources, with two of the most important being salmon and berries (Van Daele et al., 2012)**.** Bears in southwest Kodiak Island used streams with spawning salmon as fish arrived, then moved to other streams as salmon abundance and quality decreased (Barnes, 1990; Deacy, Leacock, Armstrong, & Stanford, 2016). Food shortages can also affect young survival directly and indirectly by affecting their mother (Schwartz et al., 2006b). Our results demonstrate the importance of food availability and quality for understanding young and adult survival. Although reproduction probability appeared unrelated with salmon availability (as indexed by biomass), salmon availability appeared to impact reproductive success by influencing young survival.

Further considerations for Integrated Population Model used for brown bears could include important abiotic factors for coastal brown bear populations such as weather and climate change (Mattson, 2000; Schwartz et al., 2006a; Bojarska and Selva 2012). Genetic factors including population viability, heterogeneity and plasticity, or connectivity among populations can also represent long‐term and large‐scale challenges for brown bear populations (and other mid‐to‐large‐size predators; Kamath et al., 2015). However, these have a lesser effect in the timescale that managers typically use when considering basic population models. Population variability due to stochastic and uncaptured effects can often be obtained through integration of random effects such as random year effects or spatially structured random effects; further development of our IPM would benefit from including these effects. Finally, data on annual population counts would be beneficial by providing a means to evaluate status of a population(s) throughout its range, useful for assessing management actions.

Our explicit IPM is an ecologically relevant and integrative approach to estimate animal abundance, can make other parameters identifiable, or improve their estimation. We incorporated generic and specific elements impacting population dynamics and accounted for varying sampling designs, surveys, historical datasets, and external information. Our approach can be fitted to other species or to a combination of historical data, and therefore, presents numerous applications and benefits for science and management. When looking at particular conservation objectives occurring at the interface between multiple key species or geographical areas, this approach provides a natural and intuitive framework to bring various agencies and their data together and achieve simultaneously their individual goals. The inclusion of historical data responds to the need for evaluating cultural and natural causes of variability and characterizing the overall dynamical properties of ecosystems (Swetnam, Allen, & Betancourt, 1999). The combined use of current and historical data in integrated population models can facilitate reconstruction of ecosystems or populations histories to inform management decisions.

While integrated population models are one of the most powerful methods newly added to the toolbox of managers and wildlife researchers, it is important to recognize their limitations. Namely, they require considerable data for each process modeled. Related to this issue, before modeling response variables—such as survival or detection probabilities—in function of a set of explanatory covariates, users should assess whether data sources contain enough information to statistically identify these relationships. Moreover, computation time can be long, depending on model complexity. Model selection to determine the importance of each variable should also be considered. Finally, initial modeling needs to be carefully completed to take full advantage of each dataset and correctly link all processes. Regarding the general approach we present here to provide a framework tailored to brown bears, some complementary analyses would be useful to identify its limitations. In particular, it would be useful to determine what components and data are most important. It would be useful to compare the full IPM to counts only, CMR only, litter only, and combinations of these components. Such analyses could be conducted by removing one element at a time and could provide additional information regarding which combination of components allows for the estimation of additional parameters. Because these components (counts, CMR, and litter) are not always available (or are only partially available), as in our case study, this would provide key information for managers as to which data sources are most important for their specific objectives.

The versatility of our approach would prove useful for other species. Minimal adaptations would be required for solitary large carnivores and would mostly include modifying age class and global population structures. In contrast, adaptations for social species would require accounting for density‐dependent processes, social interactions, and dependencies in detections. Further, the general population model could be modified for more or less structured systems. We recommend that spatially explicit data and components be included (such as a conditional autoregressive component to the count model, or the inclusion of an adapted spatially explicit capture–recapture model) when available. Provided data are available, and the structure of the underlying ecological models for each dataset are well understood; a wide range of species and ecosystems could be studied using variations of our modeling approach.

## CONFLICT OF INTEREST

None declared.

## DATA ACCESSIBILITY

Complete Kodiak bear data for case study have been made available on Open Science Framework (osf.io/4u28k).

## AUTHOR CONTRIBUTIONS

All authors participated to the conception and design of the work, as well as data collection. FB and JLB performed data analyses. FB, JLB, LJVD, NJS, DG, and GH interpreted the results and wrote the manuscript. All authors contributed to the final critical reviews.
