# Supplementary material for: Using multiple data types and integrated population models to improve our knowledge of apex predator population dynamics
Source: Ecol Evol. 2017 Oct 11;7(22):9531–43. doi: 10.1002/ece3.3469 (PMC5696435; doi:10.1002/ece3.3469)
Supplement: Supplementary file 1 [file ECE3-7-9531-s001.docx]

| **Category** | **Factors** | **Symbol** |
| --- | --- | --- |
| Biological | Age |  |
|  | Sex |  |
|  | Litter size |  |
|  | Mother’s age |  |
|  | Presence of dependent young |  |
|  | Age of first reproduction |  |
| Ecological | Food availability / Salmon stream density |  |
| Anthropogenic | Harvest / Hunting |  |

**Appendix S1.** Model for the population structure underlying brown bear counts, with link between considered factors and relevant parameters and age-sex groups. *With φ: survival probabilities, r: reproductive probability, L: litter size and ρ: reproductive rate.*
