# Supplementary material for: Using multiple data types and integrated population models to improve our knowledge of apex predator population dynamics
Source: Ecol Evol. 2017 Oct 11;7(22):9531–43. doi: 10.1002/ece3.3469 (PMC5696435; doi:10.1002/ece3.3469)
Supplement: Supplementary file 2 [file ECE3-7-9531-s002.docx]

**Appendix S2.** JAGS code for the general integrated population model

model{

# Global population model

# Initialization (i.e. year 1)

Ncubs_M[1] ~ dpois(lambda.Ncubs_M) # Initial number of male cubs

Nyear_M[1] ~ dpois(lambda.Nyear_M) # Initial number of yearlings

Ndep_M[1] ~ dpois(lambda.Ndep_M) # Initial number of Dependent youngs

Nsub_M[1] ~ dpois(lambda.Nsub_M) # Initial number of Subadults

Nad_M[1] ~ dpois(lambda.Nad_M) # Initial number of adults

Ncubs_F[1] ~ dpois(lambda.Ncubs_F) # Initial number of female cubs

Ncubs[1]<- Ncubs_M[1] + Ncubs_F[1] # Initial number of Total number of cubs

Nyear_F[1] ~ dpois(lambda.Nyear_F) # Initial number of yearlings

Ndep_F[1] ~ dpois(lambda.Ndep_F) # Initial number of Dependent youngs

Nsub_F[1] ~ dpois(lambda.Nsub_F) # Initial number of Subadults

Nad_F[1] ~ dpois(lambda.Nad_F) # Initial number of adults

lambda.Ncubs_M ~ dgamma(0.001,0.001)

lambda.Nyear_M ~ dgamma(0.001,0.001)

lambda.Ndep_M ~ dgamma(0.001,0.001)

lambda.Nsub_M ~ dgamma(0.001,0.001)

lambda.Nad_M ~ dgamma(0.001,0.001)

lambda.Ncubs_F ~ dgamma(0.001,0.001)

lambda.Nyear_F ~ dgamma(0.001,0.001)

lambda.Ndep_F ~ dgamma(0.001,0.001)

lambda.Nsub_F ~ dgamma(0.001,0.001)

lambda.Nad_F ~ dgamma(0.001,0.001)

# Reproductive rate

for (t in 2:nyear) {

# Abundance for males

Nyear_M.bH[t] ~ dbin(phicubs_M[t-1],Ncubs_M[t-1]) # Cubs that survived become yearlings

Ndep_M_surv[t] ~ dbin(phidep_M[t-1],Ndep_M[t-1]) # Dependent youngs that survived for another year

Ndep_M_to_sub[t] ~ dbin(transrate_dep_M[t-1],Ndep_M_surv[t]) # Out of surviving dependent youngs, some will become Subadults

Ndep_M_to_dep[t] <- Ndep_M_surv[t] - Ndep_M_to_sub[t] # The rest will stay dependent youngs

Nyear_M_to_dep[t] ~ dbin(phiyear_M[t-1],Nyear_M[t-1]) # Yearlings that survived to become dependent youngs

Ndep_M.bH[t] <- Ndep_M_to_dep[t] + Nyear_M_to_dep[t] # Dependent youngs staying dependent + Yearlings that survived to become dependent youngs

Nsub_M_surv[t] ~ dbin(phisub_M[t-1],Nsub_M[t-1]) # Subadults that survived for another year

Nsub_M_to_ad[t] ~ dbin(transrate_sub_M[t-1],Nsub_M_surv[t]) # Out of surviving Subadults, some will become Adults

Nsub_M_to_sub[t] <- Nsub_M_surv[t] - Nsub_M_to_ad[t] # The rest will stay Subadults

Nsub_M.bH[t] <- Nsub_M_to_sub[t] + Ndep_M_to_sub[t] # Subadults staying subadults + Dependent youngs that survived to become subadults

Nad_M_surv[t] ~ dbin(phiad_M[t-1],Nad_M[t-1]) # Surviving adults

Nad_M.bH[t] <- Nad_M_surv[t] + Nsub_M_to_ad[t] # Surviving adults + subadults becoming adults

# Abundance for females

Nyear_F.bH[t] ~ dbin(phicubs_F[t-1],Ncubs_F[t-1]) # Cubs that survived become yearlings

Ndep_F_surv[t] ~ dbin(phidep_F[t-1],Ndep_F[t-1]) # Dependent youngs that survived for another year

Ndep_F_to_sub[t] ~ dbin(transrate_dep_F[t-1],Ndep_F_surv[t]) # Out of surviving dependent youngs, some will become Subadults

Ndep_F_to_dep[t] <- Ndep_F_surv[t] - Ndep_F_to_sub[t] # The rest will stay dependent youngs

Nyear_F_to_dep[t] ~ dbin(phiyear_F[t-1],Nyear_F[t-1]) # Yearlings that survived to become dependent youngs

Ndep_F.bH[t] <- Ndep_F_to_dep[t] + Nyear_F_to_dep[t] # Dependent youngs staying dependent + Yearlings that survived to become dependent youngs

Nsub_F_surv[t] ~ dbin(phisub_F[t-1],Nsub_F[t-1]) # Subadults that survived for another year

Nsub_F_to_ad[t] ~ dbin(transrate_sub_F[t-1],Nsub_F_surv[t]) # Out of surviving Subadults, some will become Adults

Nsub_F_to_sub[t] <- Nsub_F_surv[t] - Nsub_F_to_ad[t] # The rest will stay Subadults

Nsub_F.bH[t] <- Nsub_F_to_sub[t] + Ndep_F_to_sub[t] # Subadults staying subadults + Dependent youngs that survived to become subadults

Nad_F_surv[t] ~ dbin(phiad_F[t-1],Nad_F[t-1])

Nad_F.bH[t] <- Nad_F_surv[t] + Nsub_F_to_ad[t] # Surviving adults + subadults becoming adults

# Reproductive rate

N_reprod_F[t] ~ dbin(p_reprod[t-1],Nad_F_surv[t]) # Number of breeding females, depending on the abundance of adult female that survived and the probability that those surviving bred during the previous season #/!\# Right now, we consider only females that survived the previous year (and during the subsequent winter), and reproduced during the previous year (because female dying in the winter would involve cubs dying/not being born)

Ncubs.bH[t] ~ dpois(lambda.Ncubs[t]) # Total number of cubs produced

lambda.Ncubs[t] <- N_reprod_F[t] * Lsize[t]

Ncubs_M.bH[t] ~ dbin(Lsexratio[t-1],Ncubs.bH[t]) # New male cubs produced

Ncubs_F.bH[t] <- Ncubs.bH[t] - Ncubs_M.bH[t] # New female cubs produced

### Harvest impact ###

# Nx refers to the number of individuals at the beginning of the year t, while harvest refers to individuals that died during year t (but before survey Y[t,o])

# If that's not the case, adapt accordingly.

# We are making sure that we are not harvesting more than there is in the population...

# Other possible formulation, e.g. for prediction of possible harvest, would be: Nyear_M[t] <- Nyear_M.bH[t] * (1-%Harvest_year_M[t])

Nyear_M[t] <- max(0, Nyear_M.bH[t] - Harvest_year_M[t] ) # Cubs that survived become yearlings

Ndep_M[t] <- max(0, Ndep_M.bH[t] - Harvest_dep_M[t] ) # Dependent youngs staying dependent + Yearlings that survived to become dependent youngs

Nsub_M[t] <- max(0, Nsub_M.bH[t] - Harvest_sub_M[t] ) # Subadults staying subadults + Dependent youngs that survived to become subadults

Nad_M[t] <- max(0, Nad_M.bH[t] - Harvest_ad_M[t] ) # Surviving adults + subadults becoming adults

Nyear_F[t] <- max(0, Nyear_F.bH[t] - Harvest_year_F[t] ) # Cubs that survived become yearlings

Ndep_F[t] <- max(0, Ndep_F.bH[t] - Harvest_dep_F[t] ) # Dependent youngs staying dependent + Yearlings that survived to become dependent youngs

Nsub_F[t] <- max(0, Nsub_F.bH[t] - Harvest_sub_F[t] ) # Subadults staying subadults + Dependent youngs that survived to become subadults

Nad_F[t] <- max(0, Nad_F.bH[t] - Harvest_ad_F[t] ) # Surviving adults + subadults becoming adults

Ncubs_M[t] <- max(0, Ncubs_M.bH[t] - Harvest_cubs_M[t] ) # New male cubs produced

Ncubs_F[t] <- max(0, Ncubs_F.bH[t] - Harvest_cubs_F[t] ) # New female cubs produced

Ncubs[t] <- Ncubs_M[t] + Ncubs_F[t]

} #/t

# Population parameters estimations from marked individuals

# Reproductive rate

for(k in 1:nrecord.litter){ ####/!\ t.litter=1 <=> birth of the litter (not necessarily observed)

# litter age: 1 <=> cubs (age between 0 and 1), 2 <=> yearlings (age between 1 and 2), >=3 <=> dependent youngs (age >2)

Lsize_ind[k,1] ~ dpois(lambda.Lsize_ind[k,1])

log(lambda.Lsize_ind[k,1]) <- intercept.Lsize + m.age_Lsize*mother.age[indiv[k],year.litter[k,1]] + m.first.reprod_Lsize*first.reprod.L[indiv[k]] + m.food_Lsize*food[year.litter[k,1]] + eps.Lsize[k]

Lweaning[k,1]~dbern(p.weaning.tmp[k,1])

p.weaning.tmp[k,1] <- p.weaning[k,1]* step(Lsize_ind[k,1]-1) * step(age.litter[k,1]-3) * step(4-age.litter[k,1]) + step(Lsize_ind[k,1]-1) * step(age.litter[k,1]-5) # prob that the litters is weaned depends on instrinsic weaning prob, the fact that the litter is still alive, and that the litter at least 3 years old (no cubs, no yearlings)

# We could also simply do our loops with t.litter=1:2 -> p.weaning.tmp=0 ; t.litter=3:4 -> p.weaning.tmp=p.weaning ; t.litter>4 -> p.weaning=1

logit(p.weaning[k,1]) <- intercept.weaning + m.age.weaning*age.litter[k,1] + eps.weaning[k]

Nmale.in.litter[k] ~ dbin(Lsexratio[year.litter[k,1]],Lsize_ind[k,1]) # Number of males in the initial litter in function of litter size and sex ratio

# /!\ Modify for litters borned before beginning of study, not possible without food covariates

}

for(k in 1:(nrecord.litter-single.y.litter)){

for(t.litter in 2:nyear.litter[k]){

# Survival process youngs

# # Case: more than one year of history

logit(phiyoung_ind[k,(t.litter-1)]) <- intercept.phiyoung + m.age*age.litter[k,(t.litter-1)] + m2.age*age.litter[k,(t.litter-1)]*age.litter[k,(t.litter-1)] + m.Lsize*Lsize_ind[k,(t.litter-1)] + m.mother.age*mother.age[indiv[k],year.litter[k,(t.litter-1)]] + m.food_young*food[year.litter[k,(t.litter-1)]] + eps_young[k] # + m.sex_cubs*sex[i] ## Impacted by age, sex, litter size, mother's age and food availability/salmon stream density

Lsize_ind[k,t.litter]~dbin(phiyoung_ind[k,(t.litter-1)],Lsize_ind[k,(t.litter-1)]) #Will either fit or simulate to following year (depending on if more than 1 year, or only 2 years of history)

logit(p.weaning[k,t.litter]) <- intercept.weaning + m.age.weaning*age.litter[k,t.litter] + eps.weaning[k]

p.weaning.tmp[k,t.litter] <- p.weaning[k,t.litter]* step(Lsize_ind[k,t.litter]-1) * step(age.litter[k,t.litter]-3) * step(4-age.litter[k,t.litter]) + step(Lsize_ind[k,t.litter]-1) * step(age.litter[k,t.litter]-5) # equal to p.weaning if litter is alive and 3=< age =< 4, equal to 0 if dead or under 3, equal to 1 if alive and >=5

Lweaning[k,t.litter]~dbern(p.weaning.tmp[k,t.litter])

}

}

for(i in 1:nindiv){

# Initialization

z.ad[i,year.indiv.beginning[i]] ~ dbern(1) # phiad_ind_tmp[I,T] CORRESPONDS TO SURVIVAL PROBABILITY OF INDIVIDUAL I BETWEEN YEAR T AND T+1

Reprod_ind[i,year.indiv.beginning[i]]~dbern(p_reprod_ind_tmp[i,year.indiv.beginning[i]])

p_reprod_ind_tmp[i,year.indiv.beginning[i]] <- p_reprod_ind[i,year.indiv.beginning[i]] * p.detect_reprod * z.ad[i,year.indiv.beginning[i]] * sex[i] # sex included because we only have data about females. Reproduction data for males is a table of 0's.

logit(p_reprod_ind[i,year.indiv.beginning[i]]) <- intercept.p.reprod + m.age_reprod*age[i,year.indiv.beginning[i]] + m.pres.dep_reprod*dep.young[i,year.indiv.beginning[i]] + m.first.reprod_reprod*first.reprod[i] + m.food_reprod*food[year.indiv.beginning[i]] + eps.reprod[i,year.indiv.beginning[i]] ## Impacted by age, presence of dependent youngs, age of first reprod and food availability/salmon stream density #~ dbeta()

y.ad[i,year.indiv.beginning[i]]~dbern(phiad_ind_obs[i,year.indiv.beginning[i]]) # observed survival/death (data)

phiad_ind_obs[i,year.indiv.beginning[i]] <- 1 #Individual is obligatory alive and "detected" on its first year in the dataset

r.ad[i,year.indiv.beginning[i]]~dbern(p.recov.ad[i,year.indiv.beginning[i]]) # observed death (i.e. recovery) (data)

p.recov.ad[i,year.indiv.beginning[i]] <- p.recov*(1-z.ad[i,year.indiv.beginning[i]])

sub.indic[i,year.indiv.beginning[i]]<-step(4-age[i,year.indiv.beginning[i]]) #1 if e >= 0; 0 otherwise

# Dynamic

for(t.indiv in (year.indiv.beginning[i]+1):nyear){

Reprod_ind[i,t.indiv]~dbern(p_reprod_ind_tmp[i,t.indiv])

p_reprod_ind_tmp[i,t.indiv] <- p_reprod_ind[i,t.indiv] * p.detect_reprod * z.ad[i,t.indiv]

logit(p_reprod_ind[i,t.indiv]) <- intercept.p.reprod + m.age_reprod*age[i,t.indiv] + m.pres.dep_reprod*dep.young[i,t.indiv] + m.first.reprod_reprod*first.reprod[i] + m.food_reprod*food[t.indiv] + eps.reprod[i,t.indiv] ## Impacted by age, presence of dependent youngs, age of first reprod and food availability/salmon stream density #~ dbeta()

# Survival process adults and subadults

z.ad[i,t.indiv]~ dbern(phiad_ind_tmp[i,t.indiv-1]) # actual survival/death (inferred)

phiad_ind_tmp[i,t.indiv-1] <- phiad_ind[i,t.indiv-1] * z.ad[i,t.indiv-1]

y.ad[i,t.indiv]~dbern(phiad_ind_obs[i,t.indiv]) # observed survival/death (data)

phiad_ind_obs[i,t.indiv] <- p.detect*z.ad[i,t.indiv] + p.recov*(1-z.ad[i,t.indiv])

r.ad[i,t.indiv]~dbern(p.recov.ad[i,t.indiv]) # observed death (i.e. recovery) (data)

p.recov.ad[i,t.indiv] <- p.recov*(1-z.ad[i,t.indiv])

# Survival probabilities

logit(phiad_ind[i,t.indiv-1]) <- intercept.phiad + m.age*age[i,t.indiv-1] + m2.age*age[i,t.indiv-1]*age[i,t.indiv-1] + m.sex_ad*sex[i] + m.food_ad*food[t.indiv-1] + eps_ad[i,t.indiv] + m.subadult*sub.indic[i,t.indiv-1] ## Impacted by age, sex (sex[i]=1 if female, 0 if male), (harvest/hunting if harvest pressure available) and food availability/salmon stream density (and subadults effect: if reprod occurred)

sub.indic[i,t.indiv]<-step(4-age[i,t.indiv]) # 1 if sub, 0 if ad (step function:1 if e >= 0; 0 otherwise)

}

}

for(t in 1:(nyear-1)){

# Global adults survival probabilities (derived) from phiad_ind[i,t.indiv] (/!\ for year t, only consider individuals that were alive in year t-1), depending on if reprod

for(index.list.indiv in 1: n.indiv.yearly[t]){

phiad_global_tmp[index.list.indiv,t]<-phiad_ind_tmp[list.indiv.yearly[t,index.list.indiv],t]

zad_global_tmp[index.list.indiv,t]<-z.ad[list.indiv.yearly[t,index.list.indiv],t]

phisub_M_tmp[index.list.indiv,t] <- phiad_ind_tmp[list.indiv.yearly[t,index.list.indiv],t] * (1-sex[list.indiv.yearly[t,index.list.indiv]]) * sub.indic[list.indiv.yearly[t,index.list.indiv],t]

zsub_M_tmp[index.list.indiv,t]<-z.ad[list.indiv.yearly[t,index.list.indiv],t] * (1-sex[list.indiv.yearly[t,index.list.indiv]]) * sub.indic[list.indiv.yearly[t,index.list.indiv],t]

phiad_M_tmp[index.list.indiv,t] <- phiad_ind_tmp[list.indiv.yearly[t,index.list.indiv],t] * (1-sex[list.indiv.yearly[t,index.list.indiv]]) * (1-sub.indic[list.indiv.yearly[t,index.list.indiv],t])

zad_M_tmp[index.list.indiv,t]<-z.ad[list.indiv.yearly[t,index.list.indiv],t] * (1-sex[list.indiv.yearly[t,index.list.indiv]]) * (1-sub.indic[list.indiv.yearly[t,index.list.indiv],t])

phisub_F_tmp[index.list.indiv,t] <- phiad_ind_tmp[list.indiv.yearly[t,index.list.indiv],t] * sex[list.indiv.yearly[t,index.list.indiv]] * sub.indic[list.indiv.yearly[t,index.list.indiv],t]

zsub_F_tmp[index.list.indiv,t]<-z.ad[list.indiv.yearly[t,index.list.indiv],t] * sex[list.indiv.yearly[t,index.list.indiv]] * sub.indic[list.indiv.yearly[t,index.list.indiv],t]

phiad_F_tmp[index.list.indiv,t] <- phiad_ind_tmp[list.indiv.yearly[t,index.list.indiv],t] * sex[list.indiv.yearly[t,index.list.indiv]] * (1-sub.indic[list.indiv.yearly[t,index.list.indiv],t])

zad_F_tmp[index.list.indiv,t]<-z.ad[list.indiv.yearly[t,index.list.indiv],t] * sex[list.indiv.yearly[t,index.list.indiv]] * (1-sub.indic[list.indiv.yearly[t,index.list.indiv],t])

# Global reproduction probability (derived)

p_reprod_global_tmp[index.list.indiv,t] <- p_reprod_ind[list.indiv.yearly[t,index.list.indiv],t] * z.ad[list.indiv.yearly[t,index.list.indiv],t] * sex[list.indiv.yearly[t,index.list.indiv]]

zreprod_Fglobal_tmp[index.list.indiv,t]<- z.ad[list.indiv.yearly[t,index.list.indiv],t] * sex[list.indiv.yearly[t,index.list.indiv]]

}

phiad_global[t] <-sum(phiad_global_tmp[1: n.indiv.yearly[t],t])/max(1,sum(zad_global_tmp[1: n.indiv.yearly[t],t]))

phisub_M[t] <- sum(phisub_M_tmp[1: n.indiv.yearly[t],t])/max(1,sum(zsub_M_tmp[1: n.indiv.yearly[t],t]))

phiad_M[t] <- sum(phiad_M_tmp[1: n.indiv.yearly[t],t])/max(1,sum(zad_M_tmp[1: n.indiv.yearly[t],t]))

phisub_F[t] <- sum(phisub_F_tmp[1: n.indiv.yearly[t],t])/max(1,sum(zsub_F_tmp[1: n.indiv.yearly[t],t]))

phiad_F[t] <- sum(phiad_F_tmp[1: n.indiv.yearly[t],t])/max(1,sum(zad_F_tmp[1: n.indiv.yearly[t],t]))

p_reprod[t] <- sum(p_reprod_global_tmp[1:n.indiv.yearly[t],t])/max(1,sum(zreprod_Fglobal_tmp[1:n.indiv.yearly[t],t]))

}

for(t in 1:nyear){

# Global litter size (derived) (loop over only newly produced litters in year t ==> needs to find records k for t==year.litter[k,1])

for(k in 1:nrecord.litter){ ####/!\ t.litter=1 <=> birth of the litter (not necessarily observed)

Lsize_global_tmp[t,k] <- exp( intercept.Lsize + m.age_Lsize*mother.age[indiv[k],year.litter[k,1]] + m.first.reprod_Lsize*first.reprod.L[indiv[k]] + m.food_Lsize*food[year.litter[k,1]] + 0.5/tau.Lsize ) *step(t-year.litter[k,1])*step(year.litter[k,1]-t)

n.new.litter_tmp[t,k]<- step(t-year.litter[k,1])*step(year.litter[k,1]-t)

}

Lsize[t] <- sum(Lsize_global_tmp[t,1:nrecord.litter]) / max(1,sum(n.new.litter_tmp[t,1:nrecord.litter]))

n.new.litter[t]<- sum(n.new.litter_tmp[t,1:nrecord.litter])

}

# ------------------------------------------------------------------------------------------------------------------------------------------------------------------------- #

# Global youngs survival probabilities (derived) from phiyoung_ind[k,t.litter] depending on age (age.litter[k,t.litter])

for(k in 1:(nrecord.litter-single.y.litter)){

for(t.litter in 2:nyear.litter[k]){

phicubs_tmp[year.litter[k,(t.litter-1)],index.year.litter[year.litter[k,(t.litter-1)],k]] <- phiyoung_ind[k,(t.litter-1)] * step(age.litter[k,(t.litter-1)]-1)*step(1-age.litter[k,(t.litter-1)])

n.cubs.litter_tmp[year.litter[k,(t.litter-1)],index.year.litter[year.litter[k,(t.litter-1)],k]]<- step(age.litter[k,(t.litter-1)]-1)*step(1-age.litter[k,(t.litter-1)])

phiyear_tmp[year.litter[k,(t.litter-1)],index.year.litter[year.litter[k,(t.litter-1)],k]] <- phiyoung_ind[k,(t.litter-1)] * step(age.litter[k,(t.litter-1)]-2)*step(2-age.litter[k,(t.litter-1)])

n.year.litter_tmp[year.litter[k,(t.litter-1)],index.year.litter[year.litter[k,(t.litter-1)],k]]<- step(age.litter[k,(t.litter-1)]-2)*step(2-age.litter[k,(t.litter-1)])

phidep_tmp[year.litter[k,(t.litter-1)],index.year.litter[year.litter[k,(t.litter-1)],k]] <- phiyoung_ind[k,(t.litter-1)] * step(age.litter[k,(t.litter-1)]-3)

n.dep.litter_tmp[year.litter[k,(t.litter-1)],index.year.litter[year.litter[k,(t.litter-1)],k]]<- step(age.litter[k,(t.litter-1)]-3)

}

}

for(t in 1:(nyear-1)){

phicubs_M[t] <-sum(phicubs_tmp[t,1:n.litter.record.per.year[t]]) /max(1,sum(n.cubs.litter_tmp[t,1:n.litter.record.per.year[t]]) ) #if sex info available: adapt previous loop ## Impacted by age, sex, litter size, mother's age and food availability/salmon stream density

phiyear_M[t] <- sum(phiyear_tmp[t,1:n.litter.record.per.year[t]])/max(1,sum(n.year.litter_tmp[t,1:n.litter.record.per.year[t]]) ) #if sex info available: adapt previous loop ## Impacted by age, sex, litter size, mother's age and food availability/salmon stream density

phidep_M[t] <- sum(phidep_tmp[t,1:n.litter.record.per.year[t]])/max(1,sum(n.dep.litter_tmp[t,1:n.litter.record.per.year[t]]) ) #if sex info available: adapt previous loop ## Impacted by age, sex, litter size, mother's age and food availability/salmon stream density

phicubs_F[t] <- sum(phicubs_tmp[t,1:n.litter.record.per.year[t]])/max(1,sum(n.cubs.litter_tmp[t,1:n.litter.record.per.year[t]]) ) #if sex info available: adapt previous loop ## Impacted by age, sex, litter size, mother's age and food availability/salmon stream density

phiyear_F[t] <- sum(phiyear_tmp[t,1:n.litter.record.per.year[t]])/max(1,sum(n.year.litter_tmp[t,1:n.litter.record.per.year[t]]) ) #if sex info available: adapt previous loop ## Impacted by age, sex, litter size, mother's age and food availability/salmon stream density

phidep_F[t] <- sum(phidep_tmp[t,1:n.litter.record.per.year[t]])/max(1,sum(n.dep.litter_tmp[t,1:n.litter.record.per.year[t]]) ) #if sex info available: adapt previous loop ## Impacted by age, sex, litter size, mother's age and food availability/salmon stream density

}

# # Sex ratio with dbeta with informative prior

## mean=a/(a+b)

## variance=a*b/((a+b)^2 *(a+b+1))

#mu.Lsexratio (entered as data, from expert/literature)

#sd.Lsexratio (entered as data, from expert/literature) (directly given or following sd=sqrt(p(1-p)/n ; with p=nbmale/tot)

var.Lsexratio <- sd.Lsexratio*sd.Lsexratio

a <-(pow(mu.Lsexratio,2) * (1-mu.Lsexratio) - mu.Lsexratio*var.Lsexratio)/var.Lsexratio

b <- a*(1-mu.Lsexratio)/mu.Lsexratio

for(t in 1:nyear){

Lsexratio[t] ~ dbeta(a,b)

# Lsexratio[t] ~ dunif(0,1) #/!\ Replace with "expert" prior if available # Litter Sex ratio, defined as the total number of males in the litters over the total number of cubs

# # Observation process

# # Population (TO ADAPT DEPENDING ON SAMPLING DESIGN)

# # Here, based on repeated counts data, p.detect also estimated thanks to CMR

for(o in 1:nocc){

Ycubs_M[t,o] ~ dbin(p.detect, Ncubs_M[t])

Yyear_M[t,o] ~ dbin(p.detect, Nyear_M[t])

Ydep_M[t,o] ~ dbin(p.detect, Ndep_M[t])

Ysub_M[t,o] ~ dbin(p.detect, Nsub_M[t])

Yad_M[t,o] ~ dbin(p.detect, Nad_M[t])

Ycubs_F[t,o] ~ dbin(p.detect, Ncubs_F[t])

Yyear_F[t,o] ~ dbin(p.detect, Nyear_F[t])

Ydep_F[t,o] ~ dbin(p.detect, Ndep_F[t])

Ysub_F[t,o] ~ dbin(p.detect, Nsub_F[t])

Yad_F[t,o] ~ dbin(p.detect, Nad_F[t])

}

}

# Transition parameters

# transition rate for dep2sub

# takes into account the number of cubs per litter

# transition rate dep = nb of dep becoming subad between year t and t+1/ (total nb of dep at year t that survived to go year t+1 )

# /!\ DEP! (yearlings and newly produced cubs are not taken into account... obviously)

for(k in 1:nrecord.litter){

for(t.litter in 1:nyear.litter[k]){

# new sub = number of dep that are alive at time t, that weaned in year t, and are age>=3 (obviously... the age verification is redundant with the information provided by Lweaning, but is here as a safety)

Nnew_sub_tmp[index.year.litter.transrate[year.litter[k,t.litter],k],year.litter[k,t.litter]] <- Lsize_ind[k,t.litter] * Lweaning[k,t.litter] * step(age.litter[k,t.litter]-3)

# N_dep_t_t.one_tmp = number of dep at t-1 that are alive at time t, and are age>=3

N_dep_t_t.one_tmp[index.year.litter.transrate[year.litter[k,t.litter],k],year.litter[k,t.litter]] <- Lsize_ind[k,t.litter] * step(age.litter[k,t.litter]-3)

}

}

# 1:n.litter.record.per.year[t]

for(t in 1:(nyear-1)){

transrate_dep_est.from.litter[t] <- sum(Nnew_sub_tmp[1:n.litter.record.per.year.transrate[t+1],t+1])/ max(1,sum(N_dep_t_t.one_tmp[1:n.litter.record.per.year.transrate[t+1],t+1])) #### Alternative: ~ dunif(0,1) #

transrate_dep_M[t] ~ dunif(0,1) # <- sum(Nnew_sub_tmp[1:n.litter.record.per.year.transrate[t+1],t+1])/ max(1,sum(N_dep_t_t.one_tmp[1:n.litter.record.per.year.transrate[t+1],t+1])) #### Alternative: #### ~ dunif(0,1) #

transrate_dep_F[t] ~ dunif(0,1) # <- transrate_dep_M[t] # We assume that we don't have data on what happens to litter in terms of sex (apart from the initial composition)

}

# transition rate sub = nb of sub becoming ad between year t and t+1/ (total nb of sub at year t that survived to go year t+1 )

for(t in 1:(nyear-1)){

for(index.list.indiv in 1: n.indiv.yearly[t]){

# sub t that survived to become ad at t+1

znew_ad_M_tmp[index.list.indiv,t]<- z.ad[list.indiv.yearly[t,index.list.indiv],t] * z.ad[list.indiv.yearly[t,index.list.indiv],t+1] * (1-sex[list.indiv.yearly[t,index.list.indiv]]) *step(age[list.indiv.yearly[t,index.list.indiv],t]-4) * step(4-age[list.indiv.yearly[t,index.list.indiv],t])

znew_ad_F_tmp[index.list.indiv,t]<- z.ad[list.indiv.yearly[t,index.list.indiv],t] * z.ad[list.indiv.yearly[t,index.list.indiv],t+1] * sex[list.indiv.yearly[t,index.list.indiv]] *step(age[list.indiv.yearly[t,index.list.indiv],t]-4) * step(4-age[list.indiv.yearly[t,index.list.indiv],t])

# sub t that survived at t+1 (ad +sub)

z_t_t.one_ad_M_tmp[index.list.indiv,t]<- z.ad[list.indiv.yearly[t,index.list.indiv],t] * z.ad[list.indiv.yearly[t,index.list.indiv],t+1] * (1-sex[list.indiv.yearly[t,index.list.indiv]]) *step(4-age[list.indiv.yearly[t,index.list.indiv],t])

z_t_t.one_ad_F_tmp[index.list.indiv,t]<- z.ad[list.indiv.yearly[t,index.list.indiv],t] * z.ad[list.indiv.yearly[t,index.list.indiv],t+1] * sex[list.indiv.yearly[t,index.list.indiv]] *step(4-age[list.indiv.yearly[t,index.list.indiv],t])

}

transrate_sub_M.tmp[t] <- sum(znew_ad_M_tmp[1: n.indiv.yearly[t],t])/ max(1,sum(z_t_t.one_ad_M_tmp[1: n.indiv.yearly[t],t]))

transrate_sub_F.tmp[t] <- sum(znew_ad_F_tmp[1: n.indiv.yearly[t],t])/ max(1,sum(z_t_t.one_ad_F_tmp[1: n.indiv.yearly[t],t]))

transrate_sub_M[t] ~ dunif(0,1) # <- min(max(transrate_sub_M.tmp[t],0.001),0.999)

transrate_sub_F[t] ~ dunif(0,1) # <- min(max(transrate_sub_F.tmp[t],0.001),0.999)

}

# Priors

p.detect ~ dunif(0,1)

p.detect_reprod ~ dunif(0,1)

p.recov ~ dunif(0,1)

intercept.Lsize ~ dnorm(0,0.0001)

m.age_Lsize ~ dnorm(0,0.0001)

m.first.reprod_Lsize ~ dnorm(0,0.0001)

m.food_Lsize ~ dnorm(0,0.0001)

intercept.weaning ~ dnorm(0,0.0001)

m.age.weaning ~ dnorm(0,0.0001)

intercept.phiyoung ~ dnorm(0,0.0001)

m.age ~ dnorm(0,0.0001)

m2.age ~ dnorm(0,0.0001)

m.Lsize ~ dnorm(0,0.0001)

m.mother.age ~ dnorm(0,0.0001)

m.food_young ~ dnorm(0,0.0001)

intercept.p.reprod ~ dnorm(0,0.0001)

m.age_reprod ~ dnorm(0,0.0001)

m.pres.dep_reprod ~ dnorm(0,0.0001)

m.first.reprod_reprod ~ dnorm(0,0.0001)

m.food_reprod ~ dnorm(0,0.0001)

intercept.phiad ~ dnorm(0,0.0001)

m.sex_ad ~ dnorm(0,0.0001)

m.food_ad ~ dnorm(0,0.0001)

m.subadult ~ dnorm(0,0.0001)

for(k in 1:nrecord.litter){

eps.Lsize[k] ~ dnorm(0,tau.Lsize)

eps.weaning[k] ~ dnorm(0,tau.weaning)

eps_young[k] ~ dnorm(0,tau_young)

}

for(i in 1:nindiv){

for(t.indiv in (year.indiv.beginning[i]):nyear){

eps.reprod[i,t.indiv] ~ dnorm(0,tau.reprod)

}

for(t.indiv in (year.indiv.beginning[i]+1):nyear){

eps_ad[i,t.indiv] ~ dnorm(0,tau_ad)

}

}

sd.Lsize ~ dunif(0,5)

tau.Lsize <- 1/(sd.Lsize*sd.Lsize)

sd.weaning ~ dunif(0,5)

tau.weaning <- 1/(sd.weaning*sd.weaning)

sd_young ~ dunif(0,5)

tau_young <- 1/(sd_young*sd_young)

sd.reprod ~ dunif(0,5)

tau.reprod <- 1/(sd.reprod*sd.reprod)

sd_ad ~ dunif(0,5)

tau_ad <- 1/(sd_ad*sd_ad)

}
